# Synergistic Antitumor Potency of a Self-Assembling Cyclodextrin Nanoplex for the Co-Delivery of 5-Fluorouracil and Interleukin-2 in the Treatment of Colorectal Cancer

**DOI:** 10.3390/pharmaceutics15020314

**Published:** 2023-01-17

**Authors:** Safiye Akkın, Gamze Varan, Anıl Işık, Sibel Gökşen, Elif Karakoç, Milo Malanga, Güneş Esendağlı, Petek Korkusuz, Erem Bilensoy

**Affiliations:** 1 Department of Pharmaceutical Technology, Faculty of Pharmacy, Hacettepe University, 06100 Ankara, Turkey; 2 Department of Vaccine Technology, Vaccine Institute, Hacettepe University, 06100 Ankara, Turkey; 3 Department of Basic Oncology, Cancer Institute, Hacettepe University, 06100 Ankara, Turkey; 4 Department of Medical and Surgical Research, Institute of Health Sciences, Hacettepe University, 06100 Ankara, Turkey; 5 Department of Histology and Embryology, Faculty of Medicine, Hacettepe University, 06100 Ankara, Turkey; 6 CycloLab-Cyclodextrin Research & Development Laboratory, Organic Synthesis Laboratory, 1097 Budapest, Hungary

**Keywords:** chemoimmunotherapy, 5-Fluorouracil, Interleukin-2, cyclodextrin polymer, nanoplex, 3D cell culture, in vivo model, colon cancer

## Abstract

Chemotherapy is the most used method after surgery in the treatment of colon cancer. Cancer cells escape the recognition mechanism of immune system cells to survive and develop chemoresistance. Therefore, the use of immunotherapy in combination with chemotherapy can increase the effectiveness of the treatment. Nanoparticles have been used clinically to increase the accumulation of therapeutics in target tissues and reduce toxicity. In this paper, nanoplexes were formed via cationic cyclodextrin polymer, 5-Fluorouracil, and Interleukin-2 based on the opposite charge interaction of macromolecules without undergoing any structural changes or losing the biological activity of Interleukin-2. Anticancer activities of nanoplexes were determined in two-dimensional and three-dimensional cell culture setups. The dual drug-loaded cyclodextrin nanoplexes diffused deeper into the spheroids and accelerated apoptosis when compared with 5-FU solutions. In the colorectal tumor-bearing animal model, survival rate, antitumor activity, metastasis, and immune response parameters were assessed using a cyclodextrin derivative, which was found to be safe based on the ALT/AST levels in healthy mice. Histomorphometric analysis showed that the groups treated with the nanoplex formulation had significantly fewer initial tumors and lung foci when compared with the control. The dual drug-loaded nanoplex could be a promising drug delivery technique in the immunochemotherapy of colorectal cancer.

## 1. Introduction

Colorectal cancer (CRC) is on the rise, and it is now the third most common cancer worldwide. Moreover, despite decreasing mortality rates, it remains the second most common cancer that leads to death [1,2]. Although surgery is preferred in stages I, II and III of CRC, chemotherapy is preferred as a preventive treatment in stage II and beyond. Chemotherapy combined with radiotherapy, targeted therapy, and immunotherapy is the preferred treatment method in stages III and IV [3]. In chemotherapy, 5-flourouracil (5-FU), camptothecin, and platinum group agents are commonly used. Furthermore, a chemotherapy combination such as FOLFOX (leucovorin (LV), 5-FU and oxaliplatin), and FOLFIRI (LV, 5-FU and irinotecan) are preferred [4,5]. The existing problems of chemotherapeutic side effects, and the inability to prevent cancer progression despite chemotherapy, have focused research on different agents, different technologies, or combinations.

5-FU is an antimetabolite that interferes with normal DNA and RNA function by taking the place of the uracil base in the structure [6]. In addition to colorectal cancer, 5-FU is used in the treatment of other cancers such as stomach, pancreas, breast, and bladder [7]. Furthermore, 5-FU has been shown to have a positive immunogenic effect in the tumor microenvironment, allowing T cells to actively fight cancer by eradicating myeloid-derived suppressor cells (MDSCs) [8]. However, liver toxicity is a typical side effect of chemotherapy for colorectal cancer, and almost half of the patients receiving 5-FU have a fatty liver and an increase in liver function tests [9,10]. Another downside of using 5-FU in therapy is its rapid distribution, with a half-life of 6 to 22 min, and limited permeability [11]. 5-FU treatment is often provided through intravenous injection. The effective dosage of 5-FU in reaching the colon is reduced by its distribution to the primary organs. According to studies, more than 85% of the administered dosage of 5-FU is converted to its inactive metabolite [12]. In this regard, research is being carried out to develop nanoparticular systems for controlled drug release and to improve the effectiveness of the pharmaceuticals reaching target tissue. Additionally, nanoparticles increase drug transport into the tumor and accumulate there [13].

Interleukin-2 (IL-2) was formerly known as a “T cell growth hormone”. It was eventually revealed to stimulate the proliferation and activation of NK cells, to contribute to the transformation of T cells into effector and memory T cells, and to aid in the creation of regulatory T cells [14]. The half-life of IL-2 is seven minutes [15], which necessitates a high dosage and repeated therapy to achieve effectiveness [14,15,16]. High dosages, on the other hand, promote the release of IL-10 and TGF-β, as well as capillary leak syndrome and disruption of neutrophil function which may be due to pleiotropic effects and serious adverse effects [17,18,19]. Low-dose and long-term IL-2 administration has been found to be safe, and the number of NK cells increases [20]. When high-doses of IL-2 are used, the surface receptors of NK cells are expressed as modified for cytotoxicity [21]. In addition, repetitive lymphocyte stimulation enhances activation-induced cell death [18]. For these reasons, a variety of approaches have been developed to limit the possibility of major adverse effects and to develop IL-2-containing systems with longer half-life at low dosages and ongoing therapeutic effectiveness. These approaches include PEGylated systems, IL-2 complexes, liposomal systems, viral and plasmid vectors, and nanoparticular systems [22].

Cyclodextrins (CD) are cyclic oligosaccharides produced by enzymatic degradation of starch [23]. CD polymers are synthesized by combining CD monomers using crosslinkers. Many nanosystems have been researched utilizing cross-linked CD polymers using epichlorohydrin (EPI), a crosslinker, since the polymers have a higher drug-loading capacity than monomers [24]. CD-polymers containing quaternary ammonium groups have superior solubility, higher hemolytic activity, and lower toxicity to erythrocytes than neutral β-CD polymers crosslinked with EPI [25]. In CaCo-2 cell cytotoxicity experiments, quaternary ammonium βCD polymer and amino βCD polymer exhibit similar effects. However, on PC12 (neuron cells), quaternary ammonium βCD polymer was found to be more toxic than amino βCD polymer after 24 h [26]. Additionally, the structure of the amino β-cyclodextrin polymer facilitates the formation of complexes with the drug and provides free penetration into biomembranes due to the polyampholyte feature [27]. Guanidino βCD polymer is a novel polymer that was synthesized for use in this study.

The nanoparticular systems engage directly with cells due to their nano structure, and allow the drug they carry to accumulate in the tumor tissue [28]. Nanoplexes are self-forming nanocarrier systems formed by the electrostatic interaction between gene or drug with a polymer/delivery structure. Nanoplexes carrying drugs or genes increase the delivery of these molecules into the tumor cells [29,30].

In our previous study, 5-FU and IL-2-loaded cationic CD nanoplexes with a particle size under 100 nm were prepared, and detailed in vitro characterization was performed. The zeta potential values of these nanoplexes were measured in the range of 14–27 mV. In vitro release profiles of CD nanoplexes with drug loading capacities of 40% and 99% for 5-FU and IL-2 were observed to be more than 80% in 12 h. The structural integrity of the IL-2 in the CD nanoplexes was confirmed by SDS-PAGE analysis. The CD polymers used in the study were shown to be non-toxic on healthy L929 murine fibroblast cells. Additionally, it was observed that against CT26 murine colon carcinoma cells, drug-loaded CD nanoplexes had a higher anticancer effect than the free drug solution [31]. The aim of this study is to elucidate the anticancer activity of 5-FU and IL-2-loaded cationic CD with real-time conventional and 3D cell culture studies and to evaluate the antitumor activity, antimetastatic properties, and immune response parameters in tumor- bearing animal model. In this way, it is aimed to increase the T lymphocyte-mediated anti-cancer effect of IL-2 and to develop an effective and reliable chemoimmunotherapy approach in the treatment of colorectal cancer.

## 2. Materials and Methods

Cationic CD polymers (quaternary ammonium βCD polymer, amino βCD polymer and guanidino βCD polymer) were synthesized and characterized by the Cyclolab R&D Laboratory, Hungary. 5-FU and recombinant mouse IL-2 were purchased from Sigma-Aldrich, Germany. CT26 murine colorectal carcinoma cell line was purchased from the American Type Culture Collection (ATCC CRL-2639). Poly (2-hydroxyethyl methacrylate) (poly-HEMA) (P3932) was purchased from Sigma-Aldrich, Germany. Matrigel^®^ Basement Membrane Matrix (356234) was purchased from Corning, U.S.A. Anti-mouse CD44 antibody and anti-IL2 antibody were purchased from Abcam, U.S.A. A TUNEL (terminal deoxynucleotidyl transferase) kit (In Situ Cell Death Detection Kit, Fluorescein-11684795910) was purchased from Roche, U.S.A. Dulbecco’s modified Eagle medium (DMEM) (D5796, Sigma-Aldrich, USA), supplemented with 10% (*v*/*v*) fetal bovine serum (FBS) (F7524, Sigma-Aldrich, USA) and 1% penicillin/streptomycin (P4333, Sigma-Aldrich, USA) was used for all cell culture studies (hereinafter referred to as “complete DMEM”). All antibodies (PerCP anti-mouse CD45, BV421 anti-mouse CD3, FITC anti-mouse CD4, BV510 anti-mouse CD44 APC-Cy7 anti-mouse CD11b, PE anti-mouse Ly6C, FITC anti-mouse Ly6G) were purchased from Biolegend, U.S.A. Ultrapure water was obtained from Millipore Simplicity 185 Ultrapure Water System (Millipore, France).

Briefly, nanoplex formulations carrying active substances were prepared with a CD: 5-FU ratio of 10:1 (*w*/*w*) and a CD:IL-2 ratio of 25,000:1 (*w*/*w*) for in vitro studies [31]; also prepared with a 1:1 (*w*/*w*) and 3906:1 (*w*/*w*) ratios for in vivo studies, respectively. An amount of 1 mL of the formulation prepared for in vivo study contained 0.5 mg of 5-FU, 0.5 mg of CD polymer and 640 IU of IL-2 (128 ng). The preparation process was the same as for the in vitro studies, only the amounts varied [31].

### 2.1. Cell Culture Studies

#### 2.1.1. Real-Time Cell Viability Analysis (RTCA)

Proliferation of CT26 cells was assessed by xCELLigence RTCA (ACEA, Roche Applied System, Switzerland). An amount of 5 × 10^3^ CT26 cells were seeded into 96-well plates coated with gold microelectrodes recording impedance as “cell index” every 30 min. After cell index exceeded 1.0, 5FU/IL2/CD nanoplexes, CD solutions, 5-FU solution or 5-FU+IL-2 solution with the desired dosage of 5-FU (the dose was 91.6 μM, and was estimated in our prior study) were applied during the 21st hour. The cell index was recorded for one week after administration, and the time-dependent cellular proliferation was evaluated.

#### 2.1.2. Antitumoral Activity of CD Nanoplexes on 3D Spheroid Tumor Culture

To create a 3D tumor spheroid with CT26 cells, round bottom 96-well plates were coated with a poly-HEMA solution as described before [31]. After keeping the lids open under the cabinet overnight, plates were seeded at a rate of 5 × 10^3^ cells/well in 200 µL with media containing 3% *v*/*v* Matrigel^®^. The plates were then centrifuged for 10 min at 10,000 rpm. The formulations were added onto the spheroids on the fifth day and cell viability was determined by WST-1 analysis after 72 h. WST-1 (10 μL) was added into the cells and then the cells were incubated for about 3 h. The absorbance was determined at 450 nm with a microplate reader, and cell viability was calculated. Complete DMEM-incubated cells were used as the control, and the viability of these groups was accepted as 100%.

#### 2.1.3. Apoptosis Assays in 3D Spheroid Tumor Culture

The TUNEL kit was used to investigate the apoptotic properties of 3D spheroids treated with 5FU/IL2/CD nanoplexes. The spheroids were then incubated for 48 h with 5FU/IL2/CD nanoplexes, 5-FU solution, IL-2 solution or culture media. The media over the spheroids was removed after incubation, and the cells were rinsed three times with pH 7.4 phosphate buffer solution (PBS). Then, 4% paraformaldehyde solution (100 µL) was added in each well. The cells were incubated at room temperature for 60 min. Following incubation, 100 µL/well of permeabilization solution (0.1% Triton X-100 in 0.1% sodium citrate) was added to the cells and the plate was placed on ice for 2 min. Following that, the cells were rinsed with PBS and the TUNEL reaction mix was applied at a rate of 50 µL/well. A commercially available TUNEL kit, containing fluorescein-conjugated dUTP and based on direct labeling of the apoptotic cell, was used. The cells were incubated in the dark for 60 min at 37 °C. Finally, the cells were examined under a microscope (Nikon Ts-2 FL) after being rinsed with PBS.

#### 2.1.4. Light Microscopy Analysis of 3D Tumor Cells in Plastic Blocks

The morphological alterations in the spheroids treated with the 5FU/IL2/CD nanoplexes were examined. In addition to drug-loaded nanoplexes, spheroids were treated with blank CD solutions, 5-FU solution, 5-FU+IL-2 solution, or cell culture media as a control group. First, the medium of the spheroids was removed and washed with PBS. An amount of 2 mL of glutaraldehyde was added to the spheroids for fixation. After 1 h of fixation on ice, the spheroids were washed with a balanced PBS and incubated with osmium tetroxide for 30 min in the dark for post-fixation. Spheroids embedded in the agar were embedded in plastic blocks after dehydration in graded alcohols and clearing with propylene oxide. One micrometer thick semi-thin sections were stained with methylene blue-azure II and qualitatively evaluated for apoptotic changes under a light microscope (DM6B, Leica, Germany).

#### 2.1.5. Immunocytochemical Analysis of 3D Spheroid Tumor Culture

The immunogenic characteristics of the spheroids were examined after the formulations were applied. CD solutions, 5FU/IL2/CD nanoplexes, 5-FU solution, 5-FU+IL-2 solution or media were applied to the spheroids. The media was removed after 48 h, spheroids were rinsed with PBS. The spheroids were transferred to well slides and fixed with 4% paraformaldehyde onto well slides for 30 min, washed with PBS and then incubated for 10 min with 0.2% Triton X-100 for permeabilization, overnight with rabbit serum for a night at +4 °C for non-specific serum blocking. The spheroids were incubated with the optimal dilution (1/100) of anti-mouse CD44 and (1/100) IL-2 primary antibodies for 1 h at room temperature. DAPI nuclear staining incubation then occurred with secondary antibody Alexa flour 488 for 30 min. The spheroid samples covered with unfading occlusion medium were evaluated for the presence of specific immunolabeling under fluorescence microscope with a digital camera attachment, in comparison with negative and positive controls.

#### 2.1.6. Assessment of Biological Activity of IL-2

The spleen was extracted from 6–8-week-old healthy male BALB-c mice through dissection. The spleen was dissected in a cell strainer with a 40 μm pore size and all splenic fragments were collected. The cells were collected using the same method as the peripheral blood mononuclear cell (PBMC) phase, with centrifugation through a Histopaque-1077 (1.077 g/mL) separation buffer. Splenocytes are the local cell population that corresponds to the PBMC phase of Histopaque-1077 in mice. The cells were initially stimulated with anti-mouse CD3 in cuvette and mixed with blank CD polymer or IL-2 solution before being seeded into the wells at a density of 1 × 10^5^ cells per well. The cells were labeled with CFSE proliferation dye before this mixing. Then the cells were analyzed by flow cytometry 72 h after seeding [32].

### 2.2. In Vivo Studies

All in vivo experiments were performed in accordance with the ethical rules for protection of animals and upon approval by the Hacettepe University Animal Experiments Ethical Committee (approval number 2017/59–06 and decision number 52338575-93). Animal experiments were performed on male BALB/c mice, 6–8 weeks of age, body weight of 20–25 g, and were housed under constant temperatures of 23 ± 2 °C, 50% humidity and filtered air.

#### 2.2.1. Safety Studies of CD Polymers

It was determined whether CD polymers cause toxicity in living organisms. Firstly, IL-2 loaded CD nanoplexes were prepared and physiological saline (SF) was used as the control group (n = 5). These nanoplexes were formed with 5000 U IL-2 and 3.9 mg CD in a 100 μL volume and a single dose was injected intravenously. After 24 h, the blood was collected and centrifuged at 2000 rpm for 10 min and the serum was obtained. Measurements of aspartate aminotransferase (AST) and alanine aminotransferase (ALT) in the serum samples were used to detect the existence of toxicity.

#### 2.2.2. Assessment of In vivo Antitumor Activity of CD Nanoplexes

The antitumor activity was evaluated with the one CD polymer determined after the safety studies. Orthotopic colon tumors were established with CT26 cells. First, a cell suspension of 2 × 10^6^ CT26 cells in 100 µL (1 mg/mL; 1:10) Matrigel^®^ was prepared. The cecum was removed, incisional scratches were created on the cecum, and then repositioned in the abdominal region. The cell suspension prepared on the cecum placed in the abdominal region was inoculated. Then, the abdominal incision site was closed using a surgical suture. To prevent dehydration in the mice following the surgical procedure, 0.5 mL of sterile 0.9% NaCl was injected subcutaneously (sc.) [4]. The tumor development process was observed for almost two weeks. The formulations were administered twice a week, for a total of four intravenous injections. 5-FU solution, 5-FU+IL-2 solution, and 5FU/IL2/CD2 nanoplex were applied as treatment groups. For the control group, only pH 7.4 PBS was given.

All groups received appropriate solution or formulation in a volume of 100 µL of PBS. There were 3.9 mg of CD polymer (found in safety studies), 5000 U of IL-2 (1 µg/mice) [33], and 1.3 mg of 5-FU (65 mg/kg dose) in 100 μL, according to the groups [34]. From the first day, the body weight of the mice (at each stage of tumor formation, injection times, and treatment termination) was recorded twice a week. After two weeks of treatment, the mice were sacrificed. Then, the spleen, mesenteric lymph node, and tumor tissues were removed and transferred to an RPMI medium containing 10% fetal bovine serum (FBS). The tissues were first divided into small pieces using a scalpel and then mechanical fragmentation was performed. Following this, enzymatic degradation was achieved by incubation in a 37 °C water bath for 1 h in a mixture of collagenase (100 U/mL), DNaseI (200 U/mL) and 10% FBS–RPMI medium. After lysis, it was passed through a 40 µm sterile strainer to obtain tissue-infiltrated immune cells. By using flow cytometry, the immunophenotyping of myeloid cells (CD45^+^CD11b^+^Ly6C^+^Ly6G^-^ monocytes and CD45^+^CD11b^+^Ly6C^dim^Ly6G^+^ neutrophils) and T lymphocytes (CD45^+^CD3^+^; further categorized according to the expression of CD4 and CD44) were examined (FACS Canto II, BD). In addition, blood samples were drawn from the mice, and the serum was obtained by centrifuging at 2000 rpm for 10 min. The serum samples were kept at −80 °C until analyzed. A flow cytometric bead array was used to measure T cell-associated cytokine levels in the sera. The analyses were performed with FACS Diva v8.0.3 (BD, U.S.A.) software.

#### 2.2.3. Histomorphometry on Cancer Nodules

Colorectal tumor tissue, liver, and lung samples were fixed with 10% formaldehyde solution, dehydrated, cleared in an automated tissue processor and embedded in paraffin on a temperature-controlled station (Leica, Wetzlar, Germany). Three to four micrometer-thick serial sections obtained on a slide microtome (Leica SM2000R, Germany) were stained with hematoxylin and eosin and quantitatively evaluated under a digital camera-attached light microscope (Leica DM6B, Germany). Briefly, the total number of microscopic tumor foci were calculated on each section, and the width (a) and length (b) of each focus was measured in µm using the LASX program. The total focal area in the liver was calculated for each sample using Formula (1) [4].

p = a/2 × b/2,(1)

where p = focal area; a = width of focus; b = length of focus.

### 2.3. Statistical Analysis

All statistical analyses were performed using Student’s *t*-test and ANOVA test using GraphPad Prism 6 and GraphPad Prism 9 (San Diego, CA, USA), and Mann–Whitney U test and Kruskal–Wallis in studies with low repetitions. *p* < 0.05 was accepted as a statistically significant difference.

Since the histopathological data obtained from the samples taken from the in vivo tumor model did not show normal distribution, multiple comparisons were performed with Kruskal–Wallis, and pairwise comparison with post-hoc Bonferroni test. The data were evaluated at a 95% confidence interval.

## 3. Results

In this scope of work, three different βCD polymers were used in preparing nanoplex systems separately, and were referred to as CD1, CD2 and CD3. Table 1 shows some chemical properties of the CD polymers.

Table 1 shows that the cationic groups of the CD1 polymer were 2.2 times higher than other polymers. In addition, the molecular weight of CD1 was 60% higher compared with CD2 and CD3. CD2 and CD3 were similar in degree of substitution, molecular weight, cross-linking ratio and cationic density.

### 3.1. Cell Culture Studies

#### 3.1.1. Real-Time Proliferation Analysis

**Figure 1:**
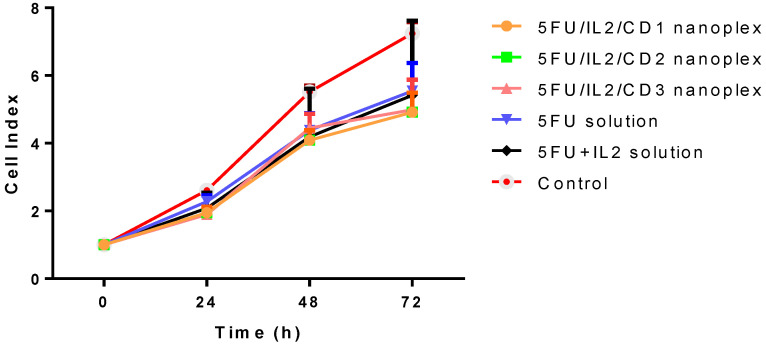
Cell index values of CT26 cells applied with 5FU/IL2/CD nanoplexes, 5-FU solution, 5-FU+IL-2 solution, and control, assessed by xCELLigence RTCA. The control group was treated with DMEM and was considered as 100% (n = 4, ± SD).

The effect of free drug and nanoplexes on CT-26 cell proliferation was determined hour by hour in real time. In addition to the 2D study conducted and shared in our publication [31], this study was planned to evaluate the change in the anticancer effect within hours. The real time proliferation rate of the control group was higher than that of experimental groups at 48 and 72 h (Figure 1).

At 48 and 72 h, the difference between 5FU/IL2/CD nanoplexes and control was significant in xCELLigence real-time cell analysis (*p* < 0.05). When compared with 5-FU solutions, however, no significant differences were found (Figure 1). When 5FU/IL2/CD nanoplexes were compared among themselves, it was found that the difference was not statistically significant (Figure 1).

**Figure 2:**
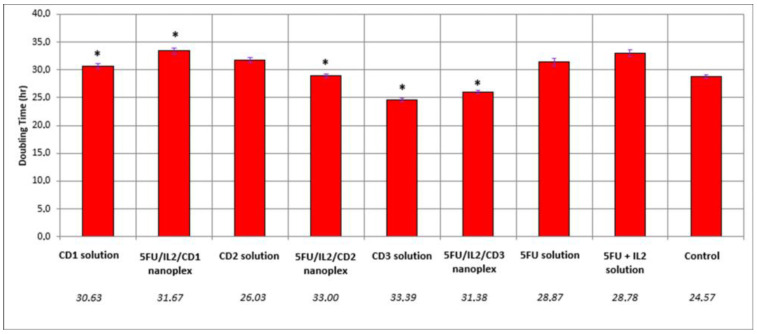
Graph of the duplication time of CT26 cells due to different formulations applied, by xCELLigence Real-Time cell analysis. The control group was treated with the DMEM and was considered as 100% (n = 4) (* *p* < 0.05 compared with 5-FU solution).

The cell duplication time was prolonged in the formulated groups compared with the control group (Figure 2). Prolongation of approximately 4 h was observed in the 5-FU solution and 5-FU+IL-2 solution relative to the control. While 2-h elongation occurred in CD2 solution, this time approached 9 h in the 5FU/IL2/CD2 nanoplex. Compared with the groups treated with 5-FU solution and 5-FU+IL-2 solution, there was a statistically significant increase in the duplication time in the groups treated with 5FU/IL2/CD1, 5FU/IL2/CD2, 5FU/IL2/CD3, CD1 solution and CD3 solution (*p* < 0.05).

#### 3.1.2. Antitumoral Activity of CD Nanoplexes on 3D Cultured Spheroid Tumor

**Figure 3:**
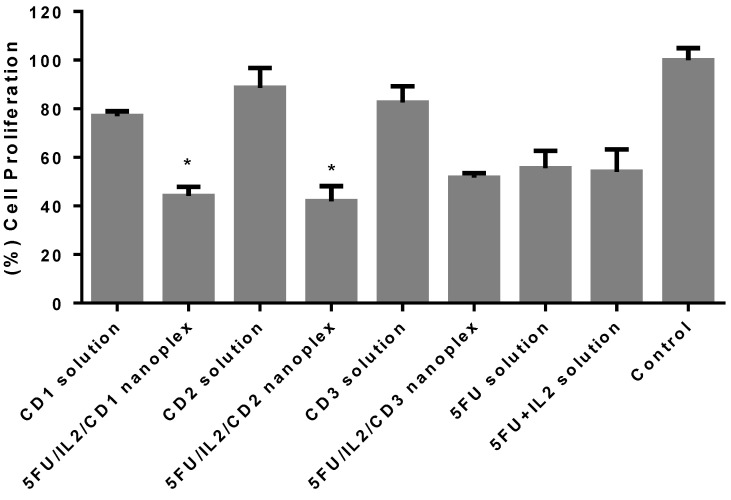
Cell viability analysis results after treatment was applied to the 3D CT-26 tumor model. The assays were performed with WST-1 after 72 h. The control group was treated with DMEM and was considered as 100% (n = 6 ± SD) (* *p* < 0.05 compared with 5-FU solution).

Anticancer activity was evaluated in a 3D model, which can better reflect the in vivo tumor structure. When the results presented in Figure 3 were evaluated statistically, 5-FU and 5-FU+IL-2 solution groups induced similar cell proliferation. According to the results, approximately 80% viability continued in the CD polymer groups. The 5FU/IL2/CD nanoplexes prepared with CD1 and CD2 polymers were more effective than the 5-FU solution (*p* < 0.05). The difference with the 5-FU solution was not significant in the 5FU/IL2/CD3 nanoplex (*p* > 0.05). Considering the average value, it can be said that the 5FU/IL2/CD2 nanoplex was the most effective of the CD nanoplexes compared with 5-FU solution.

#### 3.1.3. Apoptosis Assays of 3D Tumor Model Using TUNEL

**Figure 4:**
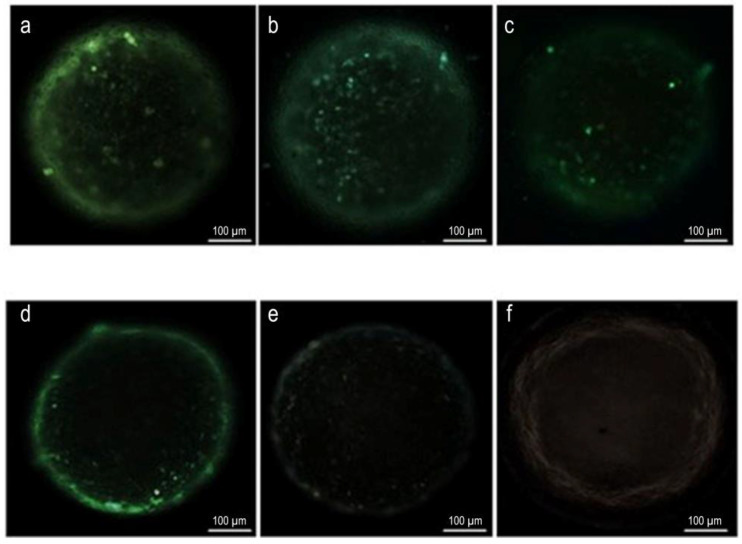
Determination of apoptosis in 3D CT26 spheroids incubated with 5FU/IL2/CD1 nanoplex (**a**), 5FU/IL2/CD2 nanoplex (**b**), 5FU/IL2/CD3 nanoplex (**c**), 5-FU solution (**d**), IL-2 solution (**e**) and culture medium (**f**). DNA strand breaks in apoptotic cells were stained green using fluoresceine-conjugated d-UTP.

The apoptotic properties of spheroids created with a 3D tumor model and applied 5FU/IL2/CD nanoplexes were determined using the TUNEL kit. The results obtained are presented in Figure 4.

According to the results, no labeling was observed in the groups treated with IL-2 solution and culture medium, respectively. The results are shown in Figure 4e,f. In the group incubated with 5-FU solution, the density of apoptotic cells was less than the groups incubated with 5FU/IL2/CD nanoplexes and the apoptotic cells were mostly on the outer surface and around the spheroid. When the formulations were examined in terms of apoptosis, all nanoplex groups caused apoptosis in the cells, however, the 5FU/IL2/CD2 nanoplex group (Figure 4b) also caused apoptosis in the cells deep inside the multilayered spheroid.

#### 3.1.4. Light Microscopy Analysis of 3D Tumor Cells on Plastic Blocks

**Figure 5:**
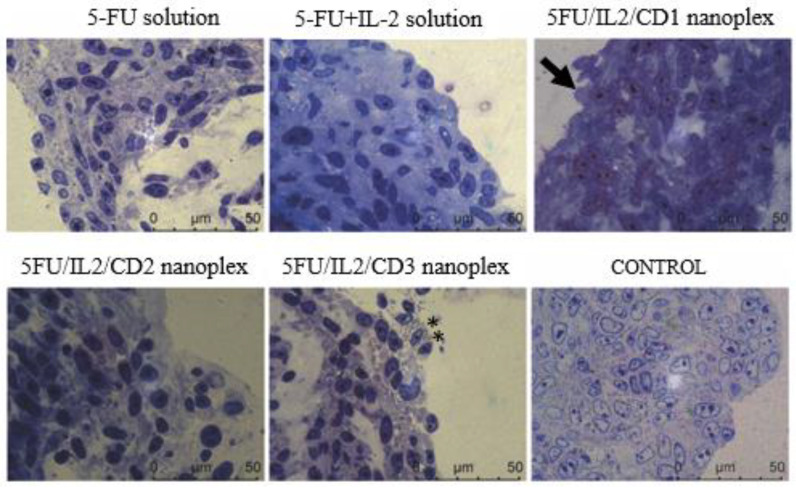
Representative micrographs of semi-thin section samples taken from the spheroids of the formulation and control groups; cells losing their integrity (**), cells undergoing apoptosis (↘).

Colon cancer cells in the control group preserved the integrity of their nuclei and cytoplasms, and the cells treated with formulations exhibited apoptotic bodies with no quantification performed (Figure 5).

#### 3.1.5. Immunocytochemical Examination in 3D Tumor Cells

**Figure 6:**
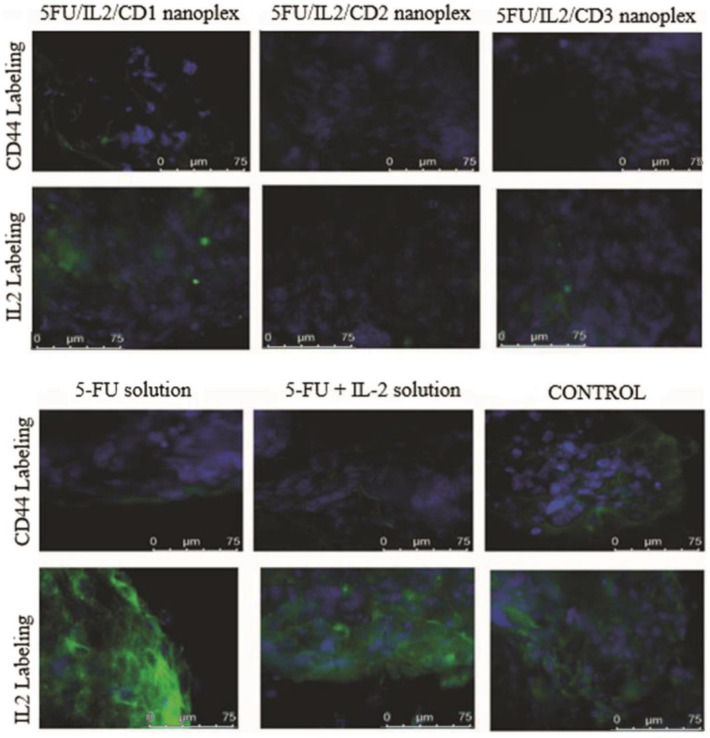
The representative micrographs of colon cancer spheroids labeled with CD44 and IL-2. Nucleus: (DAPI) ×1000.

The CT26 cells in the control group were marked with CD44 relatively more than the other formulations (Figure 6). Cells in the groups that were applied with CD2 solution, 5FU/IL2/CD2 nanoplex, CD3 solution and 5FU/IL2/CD3 nanoplex were observed to be weaker than the cells applied with 5-FU solution and 5-FU+IL-2 solution.

Colon cancer cells treated with 5-FU+IL-2 solution were marked more intensely than the cells in other groups.

#### 3.1.6. Assessment of Biological Activity of IL-2

**Figure 7:**
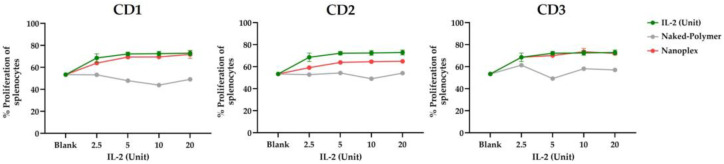
Dose dependent effect of CD polymers and naked IL-2 and IL-2 released from CD nanoplexes on splenocyte cell proliferation (n = 2, AVG ± SEM).

It was observed that IL-2 released from CD nanoplexes increased splenocyte cells at the same ratio as naked IL-2. In light of these data, IL-2 released from CD1 and CD3 nanoplexes caused proliferation in splenocytes at a similar/same rate as bare IL-2, and there was no significant change (*p* > 0.05). At all increasing doses of IL-2, splenocytes proliferated statistically significantly more than those in the control group. Even though the IL-2 loaded with CD2 polymer ten was slightly less potent for supporting splenocyte proliferation when compared with the IL-2 solution, it was still able to outperform the control group. Expectedly, IL-2 loaded nanoplex increased the splenocyte proliferation compared with bare-polymer application. Therefore, the amount of IL-2 released from the nanoplex was at appropriate concentrations to induce functional responses on immune cells. Moreover, the impact of IL-2 in IL-2-loaded nanoplex and IL-2 solution was compatible in terms of splenocyte proliferation. These results indicated that the nanoplex formulation did not interfere with functional and biological efficacy of recombinant IL-2 protein. It was observed that polymers did not negatively affect the proliferative capacity of IL-2 (Figure 7).

### 3.2. In Vivo Studies

#### 3.2.1. Safety Studies of CD Polymers

To prevent the systemic toxicity encountered in conventional chemotherapy, the safety of nanoparticular delivery systems is being investigated. An increase in ALT and AST enzymes in the blood is an indicator of liver inflammation or damage. These enzyme levels are monitored in the clinical diagnosis of the disease and in the evaluation of whether the treatments applied cause liver damage and toxicity. The PBS group was used as the control group in this study. Table 2 demonstrates that CD2 polymer is safer when considering the ALT and AST values. Consequently, the decision was undertaken to continue in vivo studies with CD2 polymer and nanoplexes.

#### 3.2.2. Antitumor Activities of CD Nanoplexes

**Figure 8:**
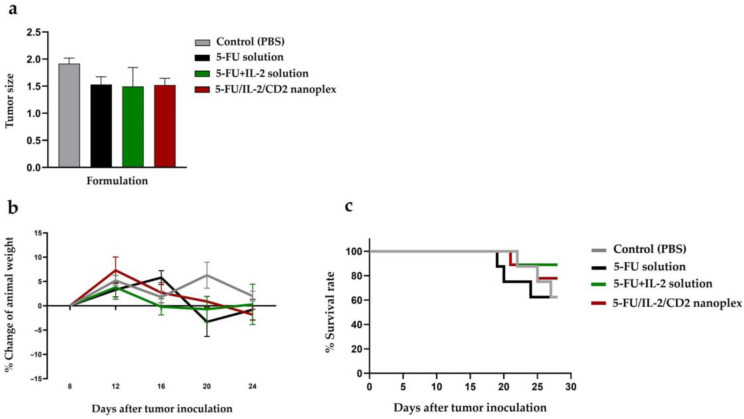
Tumor size observed at the end of the period according to the treatment applied (**a**), the change in weight over time in mice with colon cancer (**b**), and change in survival over time (**c**).

The general condition of the mice during and at the end of the treatment was evaluated in terms of parameters such as weight change. As a result of these measurements in the CT-26 tumor model that was formed in mice, a non-significant statistical difference in tumor sizes was observed in the 5FU/IL2/CD2 nanoplex groups when compared with the control group (*p* > 0.05) (Figure 8a). A non-significant statistical difference was observed in changes in the weights of the mice (*p* > 0.05) (Figure 8b). Survival rate was highest in the group treated with the 5-FU+IL-2 solution (90%). The survival rate was determined as 60% in the control group and 5-FU solution group, and approximately 80% in 5FU/IL2/CD2 nanoplex group. The addition of IL-2 to the 5-FU solution increased survival during the 28-day follow-up period compared with the 5-FU solution (Figure 8c).

**Figure 9:**
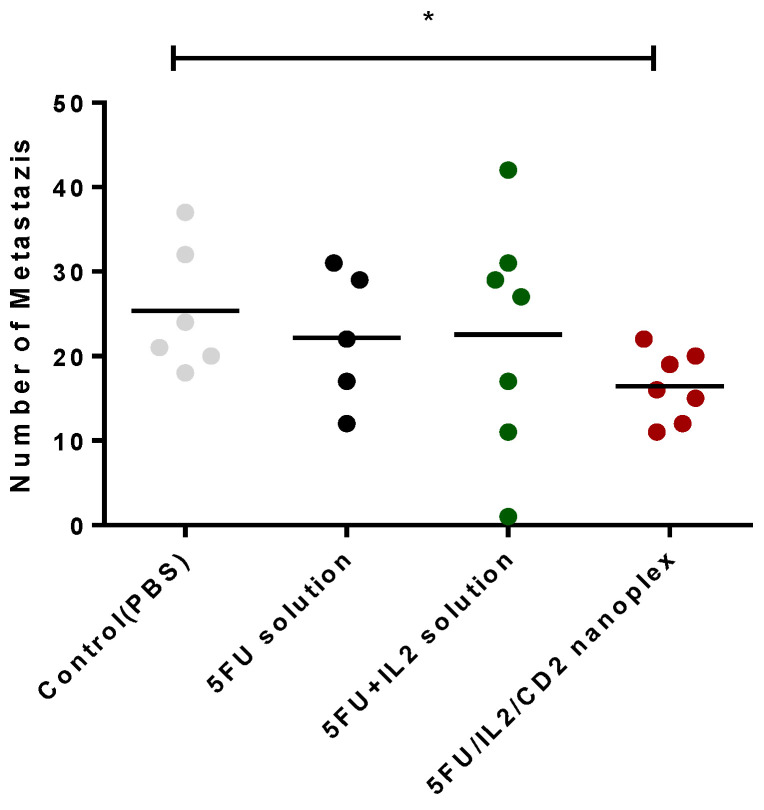
The number of metastasis foci in mice at the end of the treatment period (* *p* < 0.05).

Colorectal cancer is a type of cancer that metastasizes to the liver at a rate of 50% [35], in addition to the intra-colon or intra-rectum metastatic activity. When metastasis foci in colon and rectum were evaluated, it was observed that the number of foci was statistically lower in mice treated with 5FU/IL2/CD2 nanoplex when compared with the control group (*p* < 0.05, AVG ± SEM). The difference between nanoplex and 5-FU solution or 5-FU+IL-2 solution groups was not statistically significant (Figure 9).

**Figure 10:**
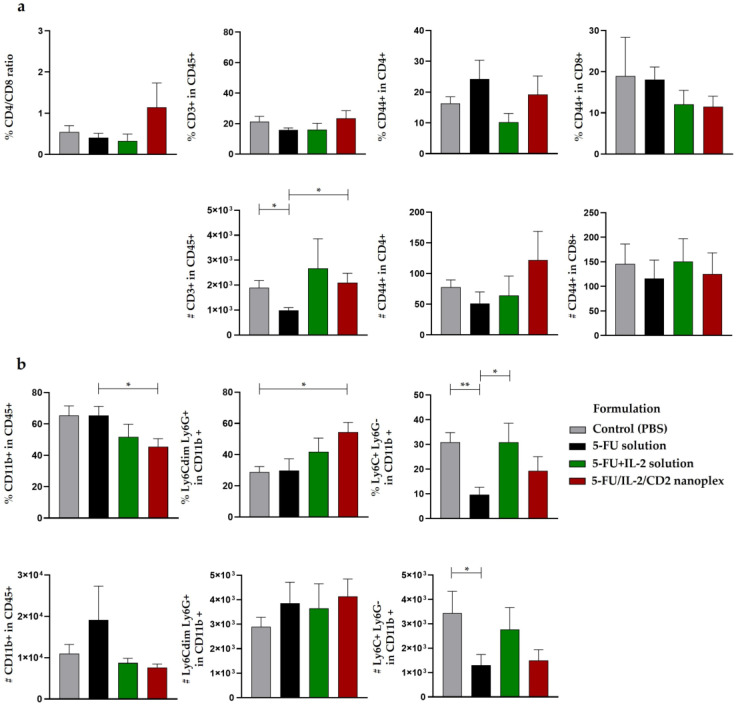

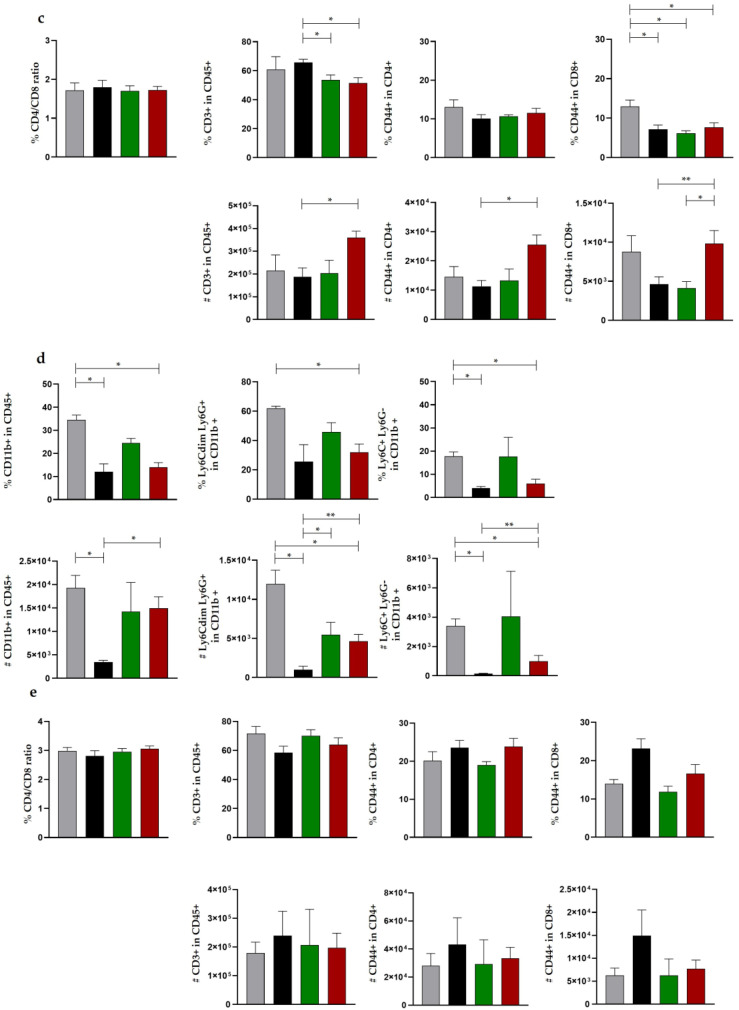
The immune cell profile in various tissues as a result of the treatments. Changes in the proportions and numbers of T lymphocytes in tumor tissue (**a**), change in myeloid cell percentages and numbers in tumor tissue (**b**), T lymphocyte numbers and percentages in the spleen (**c**), percentages and quantities of myeloid cells in the spleen (**d**), T lymphocyte numbers and percentages in the lymph node (**e**), (* *p* < 0.05, ** *p* < 0.01).

There was no statistically significant difference between the groups in the ratio of CD4^+^/CD8^+^ cells in tumor, spleen and lymph node tissues (Figure 10a,c,e).

There was no significant difference in the percentage of T cell populations in tumor tissue. However, the total number of T cells decreased in the 5-FU solution group compared with the control and 5FU/IL2/CD2 nanoplex groups. No statistically significant difference was found in the number of CD4^+^ memory T cells (CD3^+^CD4^+^CD44^+^, T_mem_) and CD8^+^T_mem_ (CD3^+^CD4^-^CD44^+^) cells in tumor tissue (Figure 10a).

While the percentage of total myeloid cells in the tumor tissue decreased in the 5FU/IL2/CD2 nanoplex group compared with the 5-FU solution group, there was no statistically significant difference between the groups in terms of cell numbers. While the percentage of the granulocyte population showed a significant increase in the 5FU/IL2/CD2 nanoplex group compared with the control group, the difference between the 5FU/IL2/CD2 nanoplex and 5-FU solution groups was not statistically significant. In addition, while the 5-FU solution group of the monocyte population decreased in percentage compared with the control and 5-FU+IL-2 solution groups, it decreased numerically only compared with the control group (Figure 10b).

The total amount of T cell populations in the spleen was decreased in the 5-FU+IL-2 solution and 5FU/IL2/CD2 nanoplex groups compared with the 5-FU solution group. When evaluated numerically, the 5FU/IL2/CD2 nanoplex group significantly increased the T cell population compared with the 5-FU solution group. While there was no significant difference in the percentage of the CD4^+^ T_mem_ cell population in the spleen, a statistically significant increase was found in the 5FU/IL2/CD2 nanoplex group compared with the 5-FU solution. The percentage of the CD8^+^ T_mem_ cell population decreased in all treatment groups compared with the control group. On the other hand, CD8+ T_mem_ cells increased numerically in the 5FU/IL2/CD2 nanoplex group compared with the 5-FU and 5-FU+IL-2 solution treatment groups (Figure 10c).

The percentage of all myeloid populations in the spleen was decreased in the 5-FU solution and 5FU/IL2/CD2 nanoplex groups compared with the control group. Numerically, the count of myeloid cells was decreased in the 5-FU solution group compared with the control and 5FU/IL2/CD2 nanoplex groups. The percentage of the granulocyte population decreased in the 5FU/IL2/CD2 nanoplex group compared with the control group. Numerically, a significant reduction was detected in all treatment groups compared with the control group. The monocyte population in the spleen decreased both in percentages and numbers in the 5-FU solution and 5FU/IL2/CD2 nanoplex groups compared with the control group. In addition, the amount of these immune cells was augmented in the 5FU/IL2/CD2 nanoplex group (Figure 10d).

When the T cell and myeloid cell populations obtained from the lymph nodes were evaluated, no statistically significant difference was observed amongst the groups both in terms of percentages and numbers (Figure 10e).

**Figure 11:**
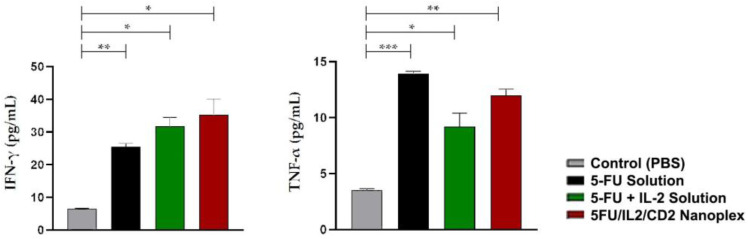
The analysis of T lymphocyte-related proinflammatory cytokine level in serum (* *p* < 0.05, ** *p* < 0.01, *** *p* < 0.001).

When the groups were evaluated in terms of IFN-γ and TNF-α anti-tumor cytokine levels, a statistically significant increase was observed in all groups when compared with the control group (*p* < 0.05). Although there was no statistically significant difference between the treated groups in terms of serum IFN-γ levels, the highest IFN-γ was determined in the groups treated with the 5-FU+IL-2 solution and 5FU/IL2/CD2 nanoplex (*p* < 0.05) (Figure 11).

#### 3.2.3. Histomorphometric Evaluation of In Vivo Cancer Nodules

**Figure 12:**
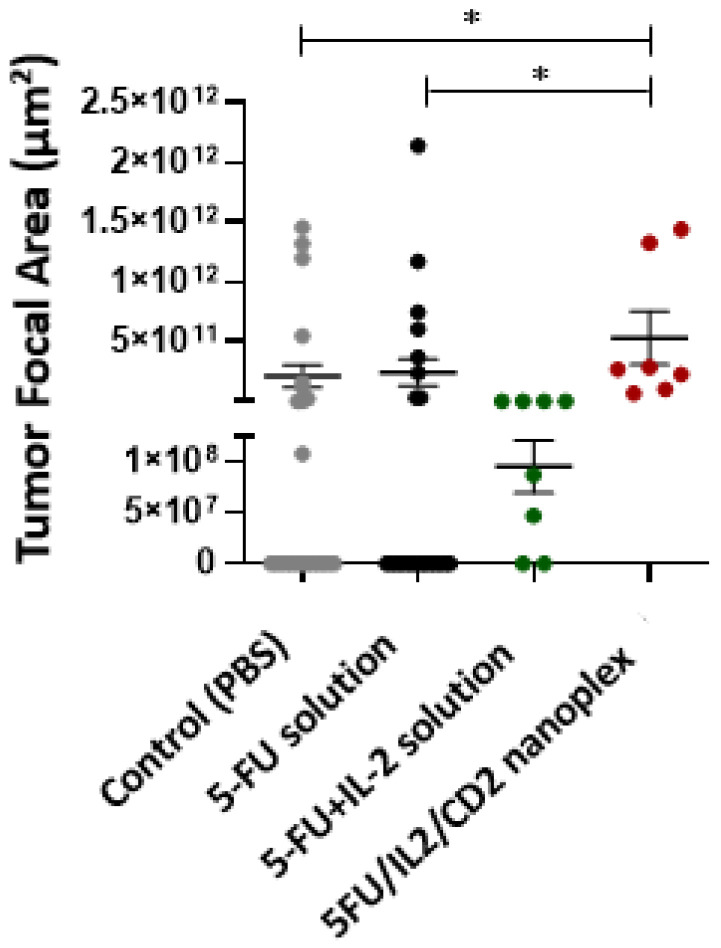
The primary tumor focal area in the treated groups with PBS, 5-FU solution, 5-FU+IL-2 solution and 5FU/IL2/CD2 nanoplex (* *p* < 0.05).

When colon tissues obtained from mice were evaluated histomorphometrically at the end of treatments, it was noted that there was statistically significantly less tumor foci developed in the group treated with the 5FU/IL2/CD2 nanoplex formulation compared with those treated with PBS and 5-FU solution (Figure 12).

**Figure 13:**
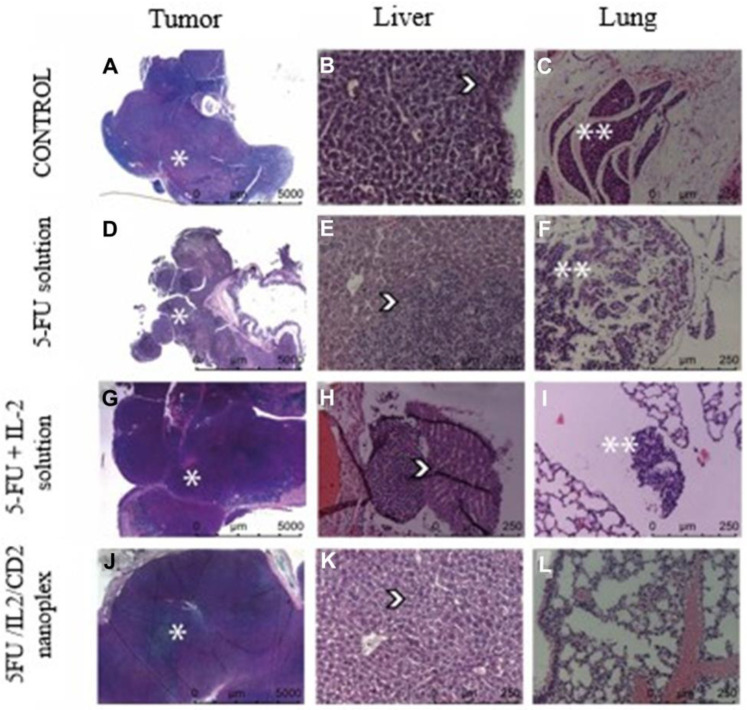
Colorectal tumor (**A**,**D**,**G**,**J**), liver (**B**,**E**,**H**,**K**) and lung (**C**,**F**,**I**,**L**) micrographs of the groups treated with PBS, 5-FU solution, 5-FU+IL-2 solution, and 5FU/IL2/CD2 nanoplex. Arrow indicates orthotopic tumor tissue and peritumoral tissue (*), metastatic tumor tissue located in the liver parenchyma (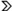), and metastatic tumor tissue located in the lung parenchyma (**).

Peritumoral tissue was present in all groups. Metastatic tumor tissue was observed in the liver in all groups. The 5FU/IL2/CD2 nanoplex group presented no lung but did present other metastases (Figure 13).

**Figure 14:**
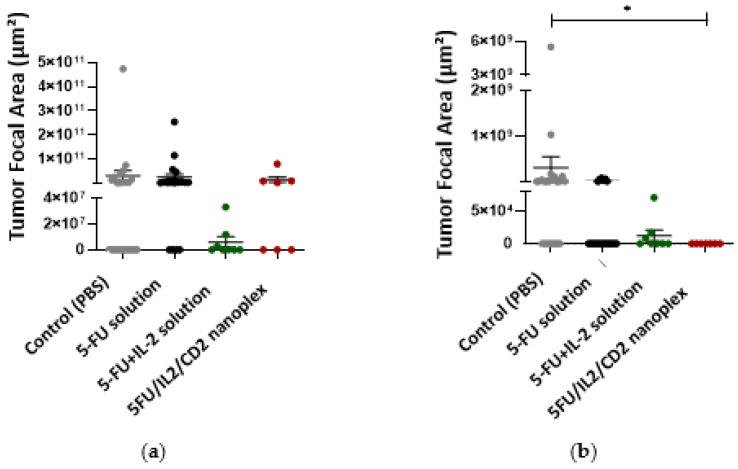
The metastatic areas (*) in liver (**a**), and lung (**b**), in treatment groups treated with PBS, 5-FU solution, 5-FU+IL-2 solution and 5FU/IL2/CD2 nanoplex (* *p* < 0.05).

There was no significant difference in the hepatic metastasis area between the PBS-treated group and the groups that received the 5-FU solution, 5-FU+IL-2 solution and 5FU/IL2/CD2 nanoplex (Figure 14a). However, when metastatic foci in the lung were analyzed, the metastatic focus area was significantly reduced in the 5FU/IL2/CD nanoplex applied group compared with the PBS applied group (Figure 14b).

## 4. Discussion

### 4.1. Cell Culture Studies

#### 4.1.1. xCELLigence Real-Time Cell Analysis

Real-time cell viability analysis was used to determine the appropriate concentration and appropriate treatment time in real time by evaluating the time-dependent effect of active substances on cells [36,37]. Real-time cell viability analysis was performed with the IC_50_ dose of 5-FU in CT26 cells calculated with the data obtained from 2D cell culture studies. When the findings of the real-time cell proliferation assay were compared with the control group, the nanoplexes prepared with each of the three CD derivatives significantly reduced cell proliferation at the end of the 48th hour. However, no significant difference was observed when nanoplex formulations were compared with solutions containing 5-FU. In conventional cell culture, in our previous study, we observed that the 5FU/IL2/CD1 nanoplex was statistically significantly more cytotoxic than 5-FU solution and 5-FU+IL-2 solution at the end of 24 and 48 h [31].

#### 4.1.2. Antitumoral Activity of CD Nanoplexes on 3D Spheroid Tumor Culture

After conventional cell culture studies, the efficiencies of the formulations were determined in the 3D cell culture method. The tumor model was successfully formed [38]. Furthermore, when the findings presented in the study were compared with the results of the conventional 2D cell culture (viability at 20% for nanoplexes), the effect of nanoplexes on cell proliferation was less in 3D cell culture (viability at 40% for nanoplexes). This was an expected result, given the structure of the in vitro 3D spheroid. In conventional cell culture studies, cells are planted as a monolayer on cell culture plates, and it is possible to treat entire cells with formulations applied on the cells. However, when the 3D structure is formed, the cells adhere to each other rather than the culture vessel and form a layered structure. Consequently, the exposure time of each cell to the formulation varies. In 3D spheroids, it is more difficult to reach the cells in the inner layers than 2D models. Despite all this, the difference between the CD nanoplex and 5-FU solution, which was not observed in 2D cells, formed in the 3D model [31]. For this reason, 3D cell culture studies are more effective methods by which to mimic in vivo models in vitro. Looking at the literature, the efficacy of 5-FU loaded chitosan nanoparticles was evaluated on HCT-116 cells by Smith et al. While the nanoparticle was found to be statistically effective at 7 μM 5-FU content in 2D cell culture, compared to 5-FU solution, this was observed at 15 μM 5-FU content in the 3D model [39]. In another study, it was stated that 5-FU loaded PLGA nanoparticles were more effective in the HT-29 spheroid model than 5-FU solution [40]. In our 2D and 3D cell culture study, 5FU/IL2/CD1 and 5FU/IL2/CD2 nanoplexes were more effective than 5-FU solution, while the 5FU/IL2/CD3 nanoplex had similar activity as 5-FU solution. The identification and observation of a more effective nanoparticle than drug solution and the similar effect of an exponential increase were also found in our study.

#### 4.1.3. Apoptosis Assays of 3D Tumor Model Using TUNEL

TUNEL shows the presence of apoptosis in cells and tissues at the DNA level. After the enzymatic processes, the DNA chain breaks are determined qualitatively by fluorescent staining. Apoptosis is programmed cell death, a natural process that takes place in the physiological process of cells and in pathological events. The elimination of tumor cells is also one of these pathological processes. In the microscope images obtained in our study, it was observed that 5-FU alone induced apoptosis. It was stated by Mhaidat et al. [41] that 5-FU induces apoptosis by activating protein kinase C and Caspase-9 in colorectal cancer cells. In TUNEL analyses performed in 2D and 3D cell culture studies with colon cancer cells, it was observed that apoptosis rates were lower in 3D samples [42]. In another study, it was stated that the entry and effect of 5-FU in colon cancer cells encountered more resistance in the 3D model than in the 2D model [43]. The efficiency of 5-FU loaded nanoparticles prepared with galactosylated chitosan based on charge interaction was evaluated in liver cells. As a result, it was found that 5-FU-loaded galactosylated chitosan nanoparticles caused more apoptosis than 5-FU solution [44]. Our study determined that 5FU/IL2/CD nanoplexes created based on charge interaction caused more apoptosis in the 3D model prepared with CT26 cells. It is thought that the efficiency of nanoplexes in penetrating the multilayered 3D structure may be better compared with 5-FU solution (Figure 4). The cationic surface charge also contributes to increased transition, due to the nanostructure of the nanoplex. This cationic structure is provided by CD polymers. In addition, CD cationic polymers increase intracellular uptake and endosomal escape [45]. Endosomal escape is an important mechanism in increasing the transport and efficiency of biological materials [46].

#### 4.1.4. Light Microscopy Analysis of 3D Tumor Cells at Plastic Blocks

In the samples obtained from spheroids, epithelial cells with pleomorphic nuclei and cytoplasm were observed in the group treated with 5-FU+IL-2 solution. Pleomorphism is defined as the change in size and shape of cells or nuclei in terms of cancer cells. The membrane structures of the tumor cells were partially dissolved and exhibited an apoptotic appearance. All of the samples of the treated groups had cells undergoing apoptosis (Figure 4.) These qualitative histological findings were found to be consistent with the data obtained in real-time proliferation analysis.

In a study on solid Ehrlich carcinoma model, free 5-FU solution was more effective than 5-FU-loaded PEG-PLGA nanoparticle in histopathological findings. However, in our study, 5FU/IL2/CD2 nanoplex and 5-FU solution showed similar efficacy which could be attributed to the favorable tumoral penetration of cationic CD nanocarriers in general [47].

#### 4.1.5. Immunocytochemical Examination in 3D Tumor Cells

In vitro 3D spheroids were also studied immunocytochemically. Firstly, 3D samples were incubated with CD44 and IL-2 antibodies. Following this incubation, staining was performed with FITC-avidin and DAPI. DAPI is a fluorescent dye that selectively binds to double-stranded DNA and forms strong fluorescent DNA-DAPI complexes with high specificity. Avidin is a protein molecule and binds strongly to biotin. Based on this binding, immunoassays were performed. FITC is the abbreviation for fluorescein isothiocyanate, which has an affinity for protein. In both stainings, it was observed that the control groups were marked more intensely by an output of green fluorescence. This result showed that the life of the cells continued. Groups treated with 5-FU solution and 5-FU+IL-2 solution were less marked, whereas CD2 polymer and nanoplex showed minimal marking. 5-FU is known to induce apoptosis in colon cancer cells, and the blue markings in the cell nucleus indicated the findings of apoptosis. Compared with the 5-FU solution groups, 5FU/IL2/CD2 nanoplex was found to have a greater potential to induce apoptosis. In a study, the efficiency of the drug delivery system created by loading 5-FU into DC-exosomes was tested in CT26 cells. Similar to our study, cells in the control group exhibited diffuse and evenly distributed blue fluorescence. In the same study, blue glow and spots were detected in the cell nuclei in the group in which 5-FU solution was applied, and apoptotic findings were confirmed. 5-FU loaded DC-exosomes had a higher tendency to induce apoptosis in cells when compared with the group treated with the 5-FU solution [48].

#### 4.1.6. Assessment of Biological Activity of IL-2

It is important that IL-2 in the nanoplex can show its biological effect without deteriorating structurally. We determined that IL-2 maintained its structural integrity within the nanoplex structure; the biological activity of IL-2 has been demonstrated in cell culture studies [49] and is also reflected by the antimetastatic activity data represented in Figure 9.

### 4.2. In Vivo Studies

#### 4.2.1. Safety Studies of CD Polymers

Safety is an important parameter for all pharmaceutical excipients. In the effectiveness of drug delivery systems, the safety of polymers is the most important parameter to be evaluated. In addition, the safety of nanoparticle delivery systems is essential in order to prevent systemic toxicity that may occur in conventional chemotherapy. For CD derivatives used in drug delivery systems, α-CD, β-CD, γ-CD, sulfobutylether-β-CD, hydroxypropyl-β-CD and randomized methyl-β-CD are in FDA GRAS (generally considered safe) status. Hemolysis, liver damage, renal damage status in blood and histological data are used to evaluate the safety of polymers. The inclusion complex of SBE βCD prepared with docetaxel was found to be safer than free docetaxel in the hemolysis and vascular stimulation test in rabbits [50]. When the implant containing poly-HPβCD and ropivacaine was examined in the rat, no negative effects were observed in the samples and histopathological data [51]. In our previous study, we determined that all polymers were safe in L929 cells in vitro [31]. Within the context of in vivo studies, firstly the safety of CD polymers was determined in healthy mice. ALT (alanine aminotransferase) and AST (aspartate aminotransferase) are enzymes produced by the liver and are the primary enzymes used in the determination of liver damage and toxicity [52]. When the results were evaluated, studies were continued with the CD2 derivative, since CD1 and CD3 derivatives may cause damage to the liver and could also be considered toxic.

Quaternary ammonium βCD polymer (CD1), amino βCD polymer hydrochloride (CD2) and guanidino βCD polymer hydrochloride (CD3) were used in this study. The antimicrobial activities of polymers based on the guanidine group have been demonstrated through membrane damage by electrostatic interaction with the lipid membrane thanks to this group [53]. It is known that the amino group forms H bonds with the -OH groups of cholesterol [54]. Quaternary ammonium groups, on the other hand, have surfactant-like activity due to their more cationic structure. For example, benzalkonium chloride contains quaternary ammonium in its structure and is used in pharmaceutical products as a strong antimicrobial preservative [55]. There is a structural difference between the polymers, which may be one reason.

#### 4.2.2. Antitumor Activities of CD Nanoplexes

This study observed that there was no significant difference between the treatment groups and the control group injected with PBS when the time-dependent mice weights were examined. However, colon cancer induced by CT26 cells caused extensive intra-abdominal tumors in the mice. It has been observed that diffuse intra-abdominal tumor causes pressure on the stomach in mice. For this reason, along with the food intake of the mice, the tumor size impacts upon the change in mice weights. In a study in the literature, which included the application of IL-2 as monotherapy, immune response was observed in mice with CT26-induced colon cancer. When morphological and pathological changes were observed, no changes were detected in major tissues—heart, spleen, liver, lung, kidney—following IL-2 administration [56]. As a result of 5-FU administration, a statistically significant decrease was observed in the number of tumor-infiltrating T lymphocytes. The nanoplexing of 5-FU with IL-2 showed an increasing trend compared with the control group, while the application of 5-FU as a solution significantly increased the number of tumor-infiltrating CD3^+^. However, their functional status needs to be detailed to evaluate the anti-tumor efficacy of T lymphocytes. When we look at the immune cell compartment in the spleen numerically, nanoplex application increased the amount of CD3^+^ lymphocytes and memory T (CD4^+^ _Tmem_ and CD8^+^ _Tmem_) lymphocytes compared with the control and 5-FU+IL-2 solution groups. In addition, a statistically significant difference was found in the nanoplex group compared with the 5-FU group. In the case of cancer, as is known from the literature, most myeloid cell populations consist of MDSCs. When looking at the myeloid cell subpopulations in the spleen, 5-FU significantly reduced the number of MDSCs compared with the control, as is known in the literature [8]. While the 5-FU+IL-2 group increased the number of MDSCs, a decrease was observed in the number of MDSCs in the nanoplex group compared with the 5-FU+IL-2 group. Although IL-2 administration with 5-FU caused an increase in the MDSC population, IL-2 is one of the critical inflammatory cytokines for T lymphocyte survival, differentiation and proliferation; the use of nanoplexes may contribute to a decrease in the number of MDSCs.

#### 4.2.3. Histopathological Evaluation of In Vivo Cancer Nodules

When the 5-FU solution and the 5-FU+IL-2 solution were compared with the 5FU/IL2/CD2 nanoplex, no difference was observed in metastasis areas in the liver. It was observed that the metastasis in the lung was less in the 5FU/IL2/CD2 nanoplex formulation groups compared with the PBS applied group, but no difference was found when compared with the 5-FU solutions. On the other hand, statistically fewer foci developed in mice treated with 5FU/IL2/CD2 nanoplex compared with mice treated with only 5-FU solution. In this context, there are literature studies determining that a combination of antibodies and 5-FU dramatically inhibits tumor growth, also suggesting that metastatic activity and recurrence can be prevented with combined treatments after chemotherapy [57].

## 5. Conclusions

Regarding our in vitro studies and results, 5FU/IL2/CD nanoplexes are more effective than drug solution in the 3D tumor model. 5FU/IL2/CD2 nanoplex had similar anticancer efficacy as other nanoplexes on a 2D structure; but infiltrated more deeply on a 3D structure. When considering the duplication time of 2D colon cancer cells in RTCA, the increase in 5FU/IL2/CD2 nanoplexes was the highest compared with the 5-FU solution, 5-FU+IL-2 solution and other nanoplexes. The biological activity of IL-2 in CD nanoplexes was maintained after forming nanoplexes.

In our in vivo study results, CD polymers were either of low toxicity or non-toxic. CD2 was found to be safer on healthy mice, therefore, in vivo studies were conducted with CD2 polymer and nanoplex in colon cancer-induced mice. Survival rates were higher in groups containing IL-2. 5FU/IL2/CD2 nanoplexes achieved better histopathological values considering primary tumor foci and the mean number of metastatic foci.

A significantly lower number of metastatic foci was observed when immunochemotherapy was administered in 5FU/IL2/CD2 nanoplexes. In terms of anticancer activity, it was observed that 5-FU and IL-2, which have no clinically approved use together, work together in free solution form to provide effective treatment.

Overall, this nanoplex platform based on only charge interaction to deliver a dual therapeutic load—a chemotherapeutic and an immunotherapy agent—was demonstrated to have favorable properties and was found effective and safe in vivo. Other therapeutic and/or diagnostic cargos would be of interest to further develop this nanocarrier system. Other cancer types with clinically approved immune therapies could also be explored with CD based nanoplexes.

## Figures and Tables

**Table 1:** The chemical properties of CD polymers used in this work.

Abbreviation	Cyclodextrin Polymers	DS	MW	CLR	CD
CD1	Quaternary-Ammonium-βCD polymer HCl, cross-linked with epichlorohydrin	2.2	40,000	11	2.2
CD2	Amino-βCD polymer HCl, cross-linked with epichlorohydrin	1	25,000	10	1
CD3	Guanidino-βCD polymer HCl, cross-linked with epichlorohydrin	1	26,000	10	1

DS: average degree of substitution; MW: average molecular weight (g/mol); CLR: cross-linking ratio (mol epichlorohydrin /mol CD); CD: cationic density (cationic groups per cyclodextrin unit).

**Table 2:** The results of serum samples collected from following the intravenous administration of different polymers to healthy mice (n = 5, AVG ± SD).

	Treatment Groups			
	PBS	CD1	CD2	CD3
AST (55-352 U/L)	367 ± 2.8	748 ± 8.5	316.5 ± 4.9	586 ± 4.2
ALT (41-131 U/L)	71 ± 2.8	71 ± 1.4	53.5 ± 0.7	79 ± 2.8
cCRP (mg/dL)	<0.3	<0.3	<0.3	<0.3
AST/ALT	5.2 ± 0.1	10.5 ± 0.4	5.9 ± 0	7.4 ± 0.3

## Data Availability

Data available upon request.
